# Development of a questionnaire for problematic social networking sites use: Ensuring content validity through Delphi methodology

**DOI:** 10.1371/journal.pone.0315442

**Published:** 2025-03-10

**Authors:** Seungju Lim, Ji-Hyuk Park

**Affiliations:** 1 Department of Occupational Therapy, Graduate School, Yonsei University, Wonju, South Korea; 2 Department of Occupational Therapy, College of Software and Digital Healthcare Convergence, Yonsei University, Wonju, South Korea; National University of Sciences and Technology, PAKISTAN

## Abstract

**Objectives:**

This study aimed to develop a questionnaire to assess problematic social network service use (PSNSU) applicable to both adolescents and adults to ensure content validity.

**Methods:**

A Delphi survey methodology with a panel of 16 experts was employed from April to June 2024 to ensure the content validity of the PSNSU assessment tool. This study involved three rounds of Delphi surveys to collect both open- and closed-ended responses to the PSNSU questionnaire. Data analysis focused on calculating the content validity ratio (CVR), stability, and consensus of each item. Items that did not meet the threshold criteria were revised or removed based on expert feedback.

**Results:**

The PSNSU questionnaire initially included three domains and 59 items. Following Round 1 of the Delphi survey, three domains, 11 subdomains, and 43 items were selected. After adding and modifying the domains and items, Round 2 resulted in a questionnaire with two domains, 10 subdomains, and 42 items. In Round 3, the revised PSNSU questionnaire showed an average CVR of 0.99, a stability of 0.10, and consensus of 0.97, thus concluding the Delphi process.

**Conclusions:**

The final PSNSU questionnaire encompasses a wide range of issues related to addictive behaviors and occupational challenges. Content validity was validated through the Delphi survey, resulting in a comprehensive tool that reflects the multidimensional characteristics of PSNSU across different life stages. This tool can be used for both clinical assessments and research to better understand and address PSNSU.

## Introduction

Social Network Services (SNS) have experienced rapid growth with the advancement of the Internet [1] and have become major communication tools because they allow users to communicate and interact with each other [2]. SNS have become globally and deeply embedded in individuals’ daily lives, with over five billion people using social media worldwide in 2024, and this number is projected to increase to almost six billion by 2028 [3]. People are motivated to use SNS for social interaction, information seeking, passing time, entertainment, relaxation, and information sharing [4]. However, excessive or problematic use of SNS (PSNSU) has been identified as a significant concern, leading to adverse effects and serious social issues [5].

PSNSU is characterized by addictive behaviors such as mood modification, salience, tolerance, withdrawal, and relapse/conflict [6], which impair various aspects of users’ lives, including educational and work performance, personal relationships, and social interactions [6,7]. Additionally, it can reduce offline social networking experiences, contribute to social comparison and feelings of inferiority [8], and cause issues such as peer phubbing [9]. Given these impacts, adolescents and adults are particularly vulnerable to PSNSU issues [10,11]. Adolescents are particularly susceptible to PSNSU because of the developmental stage of their brains, which may impair impulse regulation [12,13]. Similarly, due to COVID-19, young adults have developed a lifestyle characterized by the excessive use of SNS in daily living [11,14]. As SNS permeate various aspects of everyday life for both adolescents and young adults [7,15], assessing not only the addictive aspect but also the broader negative impact on their lives is crucial for understanding PSNSU.

However, existing assessments of PSNSU often address the issue from a single dimension and fail to fully reflect its complex and multifaceted nature. For instance, some studies, such as those utilizing the well-known Bergen Social Media Addiction Scale [16], focus solely on addictive aspects by assessing six items in the one-dimensional concepts of PSNSU. The Social Media Disorder Scale [17], also primarily measures addictive behaviors without considering the broader life impacts. While the addictive aspect has been sufficiently considered in previous studies, occupational aspects, understood as those related to daily living activities such as worsening educational performance, compromised work performance, difficulties in managing physical and psychological health, and poor rest and sleep quality [7,18], along with social aspects such as relationships with others and participation in social activities [7,11], are often overlooked in assessments.

To comprehensively understand PSNSU and its impact on users’ real lives, it is crucial to organize the PSNSU assessment to include not only the addiction domain, but also occupational and social problem domains. Therefore, it is essential to incorporate a wider range of impact areas into the PSNSU assessment [19]. Specifically, the occupational problems domain should cover issues such as education, work, health management, rest, and sleep problems [20], whereas the social problems domain should include aspects such as social comparison and peer phubbing [21]. This approach highlights the need for holistic and comprehensive tools that can capture the broad spectrum of problems caused by PSNSU, encompassing both occupational and social issues.

Moreover, existing scales and questionnaires are often limited to specific age groups such as adolescents or university students, leading to a lack of comprehensive tools that address both adolescents and adults. For example, the Problematic Use of SNS Scale [22] was developed for adolescents aged 15 to 18 years, the SNS Use Disorder Test-10 [23] for university students, and the Smartphone Addiction Scale [24] and SNS Addiction Proneness Scale [25] for college students. Consequently, these tools do not provide a holistic view of PSNSU across different life stages. This division creates challenges for comprehensively understanding the state of PSNSU and developing tailored intervention strategies. Consequently, a need arises for an assessment tool that includes both adolescents and adults and accurately reflects the multidimensional characteristics of PSNSU.

This study aimed to develop the PSNSU questionnaire to assess three main domains: addiction, occupational problems, and social problems. This tool was designed for use with both adolescents and adults using the Delphi survey methodology. Specifically, this study sought to ensure the content validity of the PSNSU assessment tool by incorporating the opinions of an expert panel.

## Methods

### Study design

A Delphi survey was conducted to ensure the content validity [26] of the PSNSU assessment tool by collecting both open- and closed-ended responses to the PSNSU questionnaire from a panel of experts. The Delphi method is a structured communication technique originally developed as a systematic, interactive forecasting method that relies on a panel of experts [27]. It is commonly used to collect and distill the knowledge and opinions of experts through a series of questionnaires interspersed with controlled opinion feedback [28,29].

### PSNSU questionnaire

The preliminary PSNSU questionnaire, developed for adolescents and young adults in the Republic of Korea, was created before achieving a content validity consensus through the Delphi methodology. The questionnaire includes three domains, 11 subdomains, and 59 items. The addiction domain consists of six subdomains: mood modification (seven items), salience (five items), tolerance (seven items), withdrawal (six items), and relapse (three items). The occupational problems domain comprises three subdomains: education/work problems (five items), health management issues (six items), and rest and sleep problems (five items). The social problem domain includes three subdomains: SNS-centered social networks (three items), social comparison (five items), and peer phubbing (seven items). This structure comprehensively assesses the multifaceted aspects of PSNSU, including addiction, occupational challenges, and social problems.

### Participants

The criteria for the expert panel in this study included: 1) experts with specialized knowledge in the field of digital addiction and mental health for adolescents and adults; 2) individuals with at least five years of clinical or research experience in the relevant field (e.g., professors, psychiatrists, occupational therapists, and researchers); 3) those who could use email for approximately two months during the Delphi period; 4) individuals with a high understanding of the Delphi survey and who could actively and continuously participate in the research; and 5) those who voluntarily agreed to participate in the study. The expert panel was recruited from 06/04/2024 to 13/04/2024. Recruitment and consent for participation were obtained via email. Sixteen individuals were recruited, and provided their written informed consent. This study was approved by the Institutional Review Board of Yonsei University Mirae Campus (approval number: 1041849-202403-SB-047-01).

### Data collection

Data were collected from 04/15/2024 to 06/05/2024, exclusively from experts who consented to participate. Before starting the Delphi survey, participants’ general characteristics (age, sex, education level, current occupation, and career duration) were collected. After collecting the general information, the Delphi survey was conducted in three rounds (Table 1). In each round, responses were collected on the relevance of the domain, subdomain, and items of the preliminary PSNSU questionnaire using a 4-point Likert scale (1, very irrelevant; 4 very relevant), along with open-ended feedback on the points to be revised (S1 file). Each round took 2–3 weeks to complete.

**Table 1:** Data Collection and Research Process.

Phase	Description
Preliminary Development	Literature review Domain and item extraction from previous studies Domain and item categorization and restructuring Development of the preliminary PSNSU questionnaire
Delphi Round 1 (16 experts)	Data collection using 11 closed-ended and 1 open-ended question on the domains, and 59 closed-ended and 1 open-ended question on the items Collected relevance responses using a 4-point Likert scale (1: very irrelevant, 4: very relevant) Analyzed CVR, Stability, and Consensus based on the data Several items were removed, modified, and added
Delphi Round 2 (16 experts)	Data collection using 11 closed-ended and 1 open-ended question on the domains, and 43 closed-ended and 1 open-ended question on the items Collected relevance responses using a 4-point Likert scale (1: very irrelevant, 4: very relevant) Analyzed CVR, Stability, and Consensus based on the data Several domains and items were removed, modified, and added
Delphi Round 3 (16 experts)	Data collection using 10 closed-ended and 1 open-ended question on the domains, and 42 closed-ended and 1 open-ended question on the items Collected relevance responses using a 4-point Likert scale (1: very irrelevant, 4: very relevant) Analyzed CVR, Stability, and Consensus based on the data
Final Development	Delphi survey confirming the content validity of the PSNSU questionnaire 2 domains, 10 subdomains, and 42 items

Note: PSNSU, problematic social network service use; CVR, content validity ratio.

#### Round 1.

The first round of the Delphi survey was conducted to assess the appropriateness of PSNSU. The domain section of the Round-1 survey consisted of 11 closed-ended questions and one open-ended question on the appropriateness of the domains. Closed-ended questions were measured on a 4-point Likert scale (Table 2), while open-ended questions gathered additional feedback on the operational definitions and conceptual distinctions of the domains and subdomains. The item section included 59 closed-ended questions and one open-ended question, measured in a similar manner.

**Table 2:** Closed-ended Questions of the Addiction Domains in PSNSU Questionnaire of the Delphi Round 1.

Question	Answer
Is it appropriate to include “mood modification” within the “addictive domain”?	Strongly irrelevant 1 2 3 4 Strongly relevant
Is it appropriate to include “salience” within the “addiction domain”?	Strongly irrelevant 1 2 3 4 Strongly relevant
Is it appropriate to include “tolerance” within the “addiction domain”?	Strongly irrelevant 1 2 3 4 Strongly relevant
Is it appropriate to include “withdrawal” within the “addiction domain”?	Strongly irrelevant 1 2 3 4 Strongly relevant
Is it appropriate to include “relapse” within the “addiction domain”?	Strongly irrelevant 1 2 3 4 Strongly relevant

#### Round 2.

Based on feedback from Round 1, the operational definitions of some subdomains were clarified, and items were removed or revised for Round 2 of the Delphi survey. The domain section of the Round 2 survey included 11 closed-ended questions and one open-ended question in the domain and subdomains. The item section included 43 closed-ended questions and one open-ended question, measured in the same manner as in Round 1.

#### Round 3.

Round 3 of the Delphi survey was conducted by restructuring the domains, subdomains, and items based on the results of Round 2. The first section of the Round 3 survey included 10 closed-ended questions and one open-ended question on the domains and subdomains. The second section included 42 closed-ended questions and one open-ended question, measured in a manner similar to that in Round 1.

### Statistical analysis

The general characteristics of the experts were analyzed using descriptive statistics with SAS software. Results from all rounds of the Delphi survey were analyzed using means, standard deviations, content validity ratios (CVR), stability, and consensus based on the closed-ended response data. Means and standard deviations were calculated from the responses collected from a 4-point Likert scale. CVR is defined as (ne−N/2)/(N/2), where ne is the number of experts rating the item as essential, and *N* is the total number of experts [30]. It is appropriate when above 0.49 with a panel of 16 experts [30]. Items below the CVR threshold were removed in this study. Stability, indicating experts’ agreement on each item, was analyzed using the coefficient of variation [31]. A coefficient less than 0.5 indicates good stability [31]. Consensus is defined as the level of agreement among experts, typically measured by calculating the percentage of agreement or using statistical measures such as the interquartile range (IQR) [32]. A threshold of consensus is often set at 75% agreement [33]. In this study, items not meeting the stability and consensus thresholds were revised or removed based on open-ended comments. The Delphi method concluded when all items met the thresholds of a CVR above 0.49, stability below 0.5, and consensus above 0.75, ensuring content validity. Excel was used for analyzing the Delphi data.

## Results

### General characteristics

The general characteristics of the 16 experts included in this study are presented in Table 3. The average age of the experts was 37.81 years, with nine females (56.25%). The majority of participants held a Ph.D. degree (11 participants, 68.75%) and were employed as professors (six participants, 37.50%). The average duration of research and clinical experience in addiction and mental health was 12.19 years.

**Table 3:** General Characteristics of the Delphi Experts.

Variable	N*	%	Min	Max
Age (M** ± SD***)	37.81	8.60	30	65
30s	12	75		
40s	3	18.75		
60s	1	6.25		
**Sex**				
Male	7	43.75		
Female	9	56.25		
**Education level**				
Bachelor’s Degree	1	6.25		
Master’s Degree	1	6.25		
Ph.D. Student	3	18.75		
Ph.D. Degree	11	68.75		
**Current occupation**				
Psychiatrist	1	6.25		
Professor	6	37.50		
Researcher	5	31.25		
Occupational therapist	4	25.00		
Career duration (M** ± SD***)	12.19	8.99	5	43

*N = sample size;

**M = mean;

***SD = standard deviation,

#### Round 1 results.

Round 1 of the Delphi survey was conducted (Table 4). In the domain section of the Delphi survey, 11 domains showed an average CVR of 0.91, stability of 0.14, and consensus of 0.90. Among the three indicators, the consensus for the SNS-centered social network (0.71) and peer phubbing (0.71) were below the threshold and were removed. Open comments on the low consensus suggest the need to further specify the meanings of both subdomains. Consequently, the term “SNS-centered social network” was clarified to mean “an individual’s preference toward using SNS for maintaining and establishing relationships,” rather than “a social network centered around SNS.” Similarly, “peer phubbing” was clarified to mean “focusing on SNS over interactions with others in social situations,” rather than “ignoring interactions with others in social settings due to SNS usage.”

**Table 4:** Results of Delphi Round 1.

	Domain/ item	M *	SD**	CVR***	Stability	Consensus	Revisions based on open-ended comments
	Domain	3.72	0.49	0.91	0.14	0.90	
	Addiction domain (5 sub-domains)						
1	Mood modification	3.44	0.73	0.75	0.21	0.75	–
2	Salience	3.81	0.40	1.00	0.10	1.00	–
3	Tolerance	3.63	0.62	0.88	0.17	0.75	–
4	Withdrawal	3.88	0.34	1.00	0.09	1.00	–
5	Relapse	3.63	0.72	0.75	0.19	0.94	–
	Occupational problem domain (3 sub-domains)						
6	Education/work problems	3.88	0.34	1.00	0.09	1.00	–
7	Health management issues	3.75	0.58	0.88	0.15	1.00	–
8	Rest and sleep problems	3.88	0.34	1.00	0.09	1.00	–
	Social problem domain (3 sub-domains)						
9	SNS-centered social network	3.44	0.63	0.88	0.18	0.71	
10	Social comparison	3.88	0.34	1.00	0.09	1.00	–
11	Peer-phubbing	3.44	0.63	0.88	0.18	0.71	
	Item	3.57	0.60	0.83	0.18	0.78	
	Mood modification sub-domain						
1	I use SNS for a change of mood.	3.75	0.45	1.00	0.12	0.94	–
2	I use SNS to forget about troublesome matters.	3.56	0.63	0.88	0.17	0.75	–
3	I use SNS to reduce depression and anxiety.	3.75	0.45	1.00	0.12	0.94	–
4	I use SNS to relieve personal stress.	3.31	0.79	0.63	0.23	0.71	Removed
5	I feel comfortable while using SNS.	3.31	0.79	0.63	0.23	0.71	I use SNS to feel calm and stable.
6	I feel confident while using SNS.	3.25	0.86	0.50	0.26	0.64	Removed
7	I feel a sense of freedom while using SNS.	3.31	0.70	0.75	0.21	0.67	Removed
	Salience sub-domain						
8	I think about SNS even when I am not using it.	3.81	0.54	0.88	0.14	1.00	–
9	I repeatedly think about things I saw on SNS.	3.69	0.60	0.88	0.16	0.94	–
10	I find it difficult to resist the urge to use SNS.	3.94	0.25	1.00	0.06	1.00	–
11	I frequently want to check SNS notifications while doing other tasks.	3.63	0.72	0.75	0.19	0.94	–
12	I suddenly think about SNS even when I am concentrating on other activities.	3.50	0.52	1.00	0.14	0.71	Removed
	Tolerance sub-domain						
13	I increasingly feel the urge to use SNS.	3.31	0.87	0.75	0.26	0.71	Removed
14	No matter how much I use SNS, I feel it’s not enough.	3.63	0.62	0.88	0.17	0.75	–
15	I use SNS longer than I intend to.	3.56	0.51	1.00	0.14	0.75	–
16	I tell myself to stop using SNS, but I keep doing it.	3.38	0.89	0.75	0.25	0.75	–
17	Once I start using SNS, I can’t stop.	3.69	0.48	1.00	0.13	0.75	–
18	I spend more and more time on SNS.	3.38	0.81	0.88	0.23	0.71	Removed
19	I spend the majority of my day on SNS.	3.44	0.63	0.88	0.18	0.71	I spend more and more time on SNS.
	Withdrawal sub-domain						
20	I feel anxious and restless when I reduce or stop using SNS.	3.94	0.25	1.00	0.06	1.00	–
21	I feel irritated when I reduce or stop using SNS.	3.81	0.40	1.00	0.10	1.00	–
22	I feel a sense of panic when I stop or reduce my SNS usage.	3.44	0.89	0.50	0.25	0.69	Removed
23	I find it difficult to cope when I stop or reduce my SNS usage.	3.38	0.72	0.75	0.21	0.71	Removed
24	I get headaches when I reduce or stop using SNS.	3.63	0.62	0.88	0.17	0.75	–
25	My appetite increases or decreases when I reduce or stop using SNS.	3.56	0.63	0.88	0.17	0.75	–
	Relapse sub-domain						
26	When I reduce my SNS usage, the urge to use it again increases.	3.50	0.52	1.00	0.14	0.71	I have tried to quit using SNS but find it difficult and end up using it again.
27	I mostly fail in my attempts to quit using SNS.	3.38	0.72	0.75	0.21	0.71	When I quit SNS, I cannot resist the urge to use it again.
28	It is difficult for me to reduce my SNS usage.	3.38	0.72	0.75	0.21	0.71	After quitting SNS, I find it hard to suppress the desire to use it when I see others using it.
	Education/work problems sub-domain						
29	I sometimes miss important academic or work-related tasks because of using SNS.	3.88	0.34	1.00	0.09	1.00	–
30	My academic performance or work productivity declines due to SNS usage.	3.94	0.25	1.00	0.06	1.00	–
31	I have had trouble focusing on my studies or work because of using SNS.	3.50	0.52	1.00	0.14	0.71	Removed
32	Frequent thoughts about SNS disrupt my academic or work activities.	3.63	0.72	0.75	0.19	0.94	–
33	I don’t have time for my studies or work because I spend too much time on SNS.	3.75	0.45	1.00	0.12	0.94	–
	Health management issues sub-domain						
34	I check SNS as soon as I wake up.	2.94	1.24	0.25	0.41	0.43	Removed
35	My excessive SNS use hinders the formation of healthy habits.	3.50	0.82	0.88	0.23	0.75	–
36	I don’t put down SNS even while walking or driving.	3.38	0.96	0.38	0.28	0.50	Removed
37	I have experienced blurred vision due to excessive SNS use.	3.38	0.81	0.88	0.23	0.71	My excessive SNS use interferes with managing blurred vision and pain in my wrists/neck.
38	I feel pain in my wrists or the back of my neck from excessive SNS use.	3.38	0.81	0.88	0.23	0.71	I have missed exercises because of using SNS.
39	I feel fatigued due to excessive SNS use.	3.50	0.52	1.00	0.14	0.71	I have hastily eaten meals due to using SNS.
	Rest and sleep problem sub-domain						
40	I lack sleep because I spend too much time on SNS.	3.69	0.60	0.88	0.16	0.94	–
41	I go to bed later because of using SNS.	3.88	0.34	1.00	0.09	1.00	–
42	I don’t get enough sleep due to using SNS.	3.50	0.52	1.00	0.14	0.71	My sleep patterns become irregular because of SNS.
43	I check SNS as soon as I wake up.	3.06	1.12	0.38	0.36	0.43	Removed
44	My excessive SNS use interferes with my rest.	3.06	0.85	0.63	0.27	0.67	I lack relaxation time due to using SNS.
							New item addition: I cannot rest my body and mind properly because of SNS.
	SNS-centered social network sub-domain						
45	I meet people more through SNS than in real life.	3.88	0.34	1.00	0.09	1.00	–
46	I feel closer to my SNS friends than to my family or friends.	3.94	0.25	1.00	0.06	1.00	–
47	I feel that my SNS friends understand me better than my family or friends do.	3.94	0.25	1.00	0.06	1.00	–
	Social comparison sub-domain						
48	I feel inferior when I see others’ posts on SNS.	3.75	0.45	1.00	0.12	0.94	–
49	People on SNS seem happier than me.	3.81	0.40	1.00	0.10	1.00	–
50	I worry that my posts or comments will be ridiculed by others.	3.44	0.63	0.88	0.18	0.71	Removed
51	If my posts get few reactions, I wonder if what I posted wasn’t good.	3.69	0.48	1.00	0.13	0.75	–
52	I feel lonely when I look at my own SNS posts and comments.	3.50	0.52	1.00	0.14	0.71	Removed
	Peer phubbing sub-domain						
53	I have missed parts of conversations with friends because I was using SNS.	3.50	0.52	1.00	0.14	0.71	Removed
54	I have ignored what others were saying because I was using SNS.	3.69	0.60	0.88	0.16	0.94	–
55	I have lost important relationships due to being overly absorbed in SNS.	3.88	0.34	1.00	0.09	1.00	–
56	I don’t have time to meet friends because of SNS.	3.50	0.52	1.00	0.14	0.71	Removed
57	I spend less time with friends because of SNS usage.	3.69	0.60	0.88	0.16	0.94	–
58	I have heard complaints from friends about my excessive SNS use.	3.44	0.63	0.88	0.18	0.71	Removed
59	My use of SNS causes conflicts with my friends.	3.75	0.58	0.88	0.15	1.00	–

In the item section, 59 items showed an average CVR of 0.83, stability of 0.18, and consensus of 0.78. Among the CVR values, health management issue items 1 (0.25) and 3 (0.38), as well as rest and sleep problems item 4 (0.38), were below the threshold and were deleted. In total, 27 items were found to be below the consensus threshold. Open comments on the low consensus indicated that 17 items (mood modification items 4, 6, and 7; salience item 5; tolerance items 1 and 6; withdrawal items 3 and 4; social comparison items 3 and 5; peer phubbing items 1, 4, and 6; education/work problems item 3; health management issues items 1 and 3; and rest and sleep problems item 4 had overlapping meanings with other items within the same domain, suggesting their deletion. For the remaining 10 items (mood modification item 5; tolerance item 7; relapse items 2 and 3; health management issues items 4, 5, and 6; and rest and sleep problems items 3 and 5), it was recommended that their meanings be clarified. Additionally, it was suggested to add an item related to rest within the rest and sleep subdomains. Consequently, 17 items with overlapping meanings were deleted, 10 items had their meanings clarified, and a new item, rest and sleep problems item 5 “I cannot rest my body and mind properly due to SNS,” was added.

#### Round 2 results.

Round 2 of the Delphi survey was conducted based on the revised PSNSU questionnaire from Round 1 (Table 5). In the domain section, 11 domains showed an average CVR of 0.99, stability of 0.12, and consensus of 0.88. Among the three indicators, the consensus for SNS-centered social networks (0.71) and peer phubbing (0.71) was below the threshold, similar to that in Round 1. The open-ended comments suggested that there was no need to separate the two subdomains. Instead, it was recommended to integrate them into the social relationship and social participation problem subdomains within the occupational problem domain. Based on this feedback, a new social relationships problem subdomain was created in the occupational problem domain, operationally defined as “issues arising from the use of SNS that negatively impact the ability to maintain and establish personal relationships.” Additionally, a new participation in social activities problem subdomain was established, operationally defined as “challenges related to engaging in social activities due to prioritizing SNS use over face-to-face interactions.”

**Table 5:** Results of Delphi Round 2.

Domain/ item		M *	SD**	CVR***	Stability	Consensus	Revisions based on open-ended comments
	Domain	3.71	0.46	0.99	0.12	0.88	
	Addiction domain (5 sub-domains)						
1	Mood modification	3.56	0.51	1.00	0.14	0.75	–
2	Salience	3.75	0.45	1.00	0.12	0.94	–
3	Tolerance	3.75	0.45	1.00	0.12	0.94	–
4	Withdrawal	3.81	0.40	1.00	0.10	1.00	–
5	Relapse	3.69	0.48	1.00	0.13	0.75	–
	Occupational problem domain (3 sub-domains)						
6	Education/work problems	3.81	0.40	1.00	0.10	1.00	
7	Health management issues	3.75	0.45	1.00	0.12	0.94	–
8	Rest and sleep problems	3.81	0.40	1.00	0.10	1.00	
	Social problem domain (3 sub-domains)						
9	SNS-centered social network	3.44	0.63	0.88	0.18	0.71	–
10	Social comparison	3.75	0.45	1.00	0.12	0.94	–
11	Peer-phubbing	3.50	0.52	1.00	0.14	0.71	–
	Item	3.69	0.53	0.94	0.19	0.88	
	Mood modification sub-domain						
1	I use SNS for a change of mood.	3.63	0.81	0.88	0.22	0.94	–
2	I use SNS to forget about troublesome matters.	3.75	0.45	1.00	0.12	0.94	–
3	I use SNS to reduce depression and anxiety.	3.69	0.48	1.00	0.13	0.75	–
4	I use SNS to feel calm and stable.	3.50	0.63	0.88	0.18	0.75	–
	Salience sub-domain						
5	I think about SNS even when I am not using it.	3.69	0.48	1.00	0.13	0.75	–
6	I repeatedly think about things I saw on SNS.	3.81	0.40	1.00	0.10	1.00	–
7	I find it difficult to resist the urge to use SNS.	3.81	0.40	1.00	0.10	1.00	–
8	I frequently want to check SNS notifications while doing other tasks.	3.63	0.50	1.00	0.13	0.75	–
	Tolerance sub-domain						
9	No matter how much I use SNS, I feel it’s not enough.	3.75	0.45	1.00	0.12	0.94	–
10	I use SNS longer than I intend to.	3.81	0.40	1.00	0.10	1.00	–
11	I tell myself to stop using SNS, but I keep doing it.	3.75	0.45	1.00	0.12	0.94	–
12	Once I start using SNS, I can’t stop.	3.81	0.40	1.00	0.10	1.00	–
13	I spend more and more time on SNS.	3.50	0.52	1.00	0.14	0.71	Move to salience sub-domain
	Withdrawal sub-domain						
14	I feel anxious and restless when I reduce or stop using SNS.	3.75	0.45	1.00	0.12	0.94	–
15	I feel irritated when I reduce or stop using SNS.	3.81	0.40	1.00	0.10	1.00	–
16	I get headaches when I reduce or stop using SNS.	3.69	0.60	0.88	0.16	0.94	–
17	My appetite increases or decreases when I reduce or stop using SNS.	3.69	0.60	0.88	0.16	0.94	–
	Relapse sub-domain						
18	I have tried to quit using SNS but find it difficult and end up using it again.	3.63	0.62	0.88	0.17	0.75	–
19	When I quit SNS, I cannot resist the urge to use it again.	3.81	0.40	1.00	0.10	1.00	–
20	After quitting SNS, I find it hard to suppress the desire to use it when I see others using it.	3.44	0.81	0.88	0.23	0.75	–
	Education/work problems sub-domain						
21	I sometimes miss important academic or work-related tasks because of using SNS.	3.75	0.58	0.88	0.15	1.00	–
22	My academic performance or work productivity declines due to SNS usage.	3.75	0.58	0.88	0.15	1.00	–
23	Frequent thoughts about SNS disrupt my academic or work activities.	3.81	0.54	0.88	0.14	1.00	–
24	I don’t have time for my studies or work because I spend too much time on SNS.	3.69	0.60	0.88	0.16	0.94	–
	Health management issues sub-domain						
25	My excessive SNS use hinders the formation of healthy habits.	3.69	0.60	0.88	0.16	0.94	–
26	My excessive SNS use interferes with managing blurred vision and pain in my wrists/neck.	3.31	0.87	0.75	0.26	0.71	Removed
27	I have missed exercises because of using SNS.	3.81	0.40	1.00	0.10	1.00	–
28	I have hastily eaten meals due to using SNS.	3.75	0.45	1.00	0.12	0.94	–
	Rest and sleep problems sub-domain						
29	I lack sleep because I spend too much time on SNS.	3.88	0.34	1.00	0.09	1.00	–
30	I go to bed later because of using SNS.	3.81	0.40	1.00	0.10	1.00	–
31	My sleep patterns become irregular because of SNS.	3.88	0.34	1.00	0.09	1.00	–
32	I lack relaxation time due to using SNS.	3.94	0.25	1.00	0.06	1.00	–
33	I cannot rest my body and mind properly because of SNS.	3.81	0.40	1.00	0.10	1.00	–
	SNS-centered social networks sub-domain						
34	I meet people more through SNS than in real life.	3.56	0.73	0.75	0.20	0.75	Move to social participation sub-domain: I mainly connect with people through SNS rather than meeting them in person.
35	I feel closer to my SNS friends than to my family or friends.	3.50	0.73	0.75	0.20	0.75	Move to social relationship sub-domain: I feel closer to the relationships I form through SNS than to those with my family and friends.
36	I feel that my SNS friends understand me better than my family or friends do.	3.56	0.73	0.75	0.20	0.75	Move to social relationship sub-domain: I feel that the relationships I form through SNS understand me better than my family and friends do.
	Social comparison sub-domain						
37	I feel inferior when I see others’ posts on SNS.	3.56	0.51	1.00	0.14	0.75	Move to health management problem sub-domain
38	People on SNS seem happier than me.	3.50	0.52	1.00	0.14	0.71	Removed
39	If my posts get few reactions, I wonder if what I posted wasn’t good.	3.56	0.51	1.00	0.14	0.75	Move to health management problem sub-domain: I feel self-doubt when my posts receive few likes and comments.
	Peer phubbing sub-domain						
40	I have ignored what others were saying because I was using SNS.	3.69	0.48	1.00	0.13	0.75	Move to social relationship sub-domain
41	I have lost important relationships due to being overly absorbed in SNS.	3.69	0.48	1.00	0.13	0.75	Move to social relationship sub-domain
42	I spend less time with friends because of SNS usage.	3.50	0.63	0.88	0.18	0.75	Move to social participation sub-domain: I spend less time with my family and friends because of my SNS usage.
43	My use of SNS causes conflicts with my friends.	3.50	0.73	0.75	0.20	0.75	Move to social relationship sub-domain: I have focused more on SNS than on the conversation when talking with my family and friends.
							New item addition in social participation sub-domain: I spend less time and participate less frequently in various events, activities, and gatherings because of using SNS.

In the item section, the analysis of the 43 items (originally 59 items from Round 1, with 17 items removed and one item added) showed an average CVR of 0.94, stability of 0.19, and consensus of 0.88. The consensus for the following three items was below the threshold. First, it is recommended that tolerance item 5 be moved from the tolerance subdomain to the salience subdomain. Second, it was suggested to delete health management issue item 2, as it was difficult to interpret the item solely as a problem arising from PSNSU. Third, social comparison item 2 was recommended for deletion because of concerns regarding its appropriateness. Accordingly, these three items were either removed or deleted.

Additionally, items related to SNS-centered social networks and peer phubbing, which showed low consensus values up to Delphi Round 1, and all social comparison items, were below the threshold for consensus. The open-ended comments suggested deleting the social problem domain and moving all items within it to the occupational problem domain. Based on this feedback, all nine items (excluding the deleted social comparison item 2 due to its low consensus) included in the social problem domain were relocated. Five items (SNS-centered social network items 2 and 3 and peer phubbing items 1, 2, and 4) were moved to the social relationship problem subdomain. Two items (SNS-centered social network item 1 and peer phubbing item 3) were reassigned to the social participation problem subdomain. The remaining two items (social comparison items 1 and 3) were interpreted as having a psychosocial health management aspect and were moved to the health management problem subdomain.

Finally, the comments suggested the addition of items to measure the time and frequency of direct participation in activities and meetings as elements of social participation. Additionally, typographical and grammatical adjustments were made to improve the overall clarity based on feedback.

#### Round 3 results.

Delphi Round 3 was conducted based on the revised PSNSU questionnaire from Round 2 (Table 6). The analysis of the revised two domains and 10 subdomains showed an average CVR of 1.00, stability of 0.10, and consensus of 0.98, all meeting the threshold criteria. The analysis of the 42 items (43 items from Round 2, two items removed, and one item added) showed an average CVR of 0.99, a stability of 0.10, and a consensus of 0.97, all meeting the threshold criteria. Consequently, all domains and items satisfied the conditions of a CVR of 0.49 or higher, stability of 0.5 or lower, and consensus of 0.75 or higher, concluding the Delphi process. The CVR, stability, and consensus for all the Delphi rounds are listed in Table 7. The final questionnaire comprised 2 domains, 10 subdomains, and 42 items (S1 Table).

**Table 6:** The Results of Delphi Round 3.

Domain/ items		M *	SD**	CVR***	Stability	Consensus
	Domain	3.81	0.40	1.00	0.10	0.98
	Addiction domain (5 sub-domains)					
1	Mood modification	3.75	0.45	1.00	0.12	0.94
2	Salience	3.81	0.40	1.00	0.10	1.00
3	Tolerance	3.75	0.45	1.00	0.12	0.94
4	Withdrawal	3.81	0.40	1.00	0.10	1.00
5	Relapse	3.75	0.45	1.00	0.12	0.94
	Occupational problem domain (5 sub-domains)					
6	Education/work problems	3.88	0.34	1.00	0.09	1.00
7	Health management issues	3.88	0.34	1.00	0.09	1.00
8	Rest and sleep problems	3.88	0.34	1.00	0.09	1.00
9	Social relationship issues	3.75	0.45	1.00	0.12	0.94
10	Social participation issues	3.81	0.40	1.00	0.10	1.00
	Item	3.83	0.38	0.99	0.10	0.97
	Mood modification sub-domain					
1	I use SNS for a change of mood.	3.81	0.40	1.00	0.10	1.00
2	I use SNS to forget about troublesome matters.	3.81	0.40	1.00	0.10	1.00
3	I use SNS to reduce depression and anxiety.	3.75	0.45	1.00	0.12	0.94
4	I use SNS to feel calm and stable.	3.56	0.51	1.00	0.14	0.75
	Salience sub-domain					
5	I think about SNS even when I am not using it.	3.75	0.45	1.00	0.12	0.94
6	I repeatedly think about things I saw on SNS.	3.88	0.34	1.00	0.09	1.00
7	I find it difficult to resist the urge to use SNS.	3.75	0.45	1.00	0.12	0.94
8	I frequently want to check SNS notifications while doing other tasks.	3.81	0.40	1.00	0.10	1.00
9	I spend more and more time on SNS.	3.88	0.34	1.00	0.09	1.00
	Tolerance sub-domain					
10	No matter how much I use SNS, I feel it’s not enough.	3.75	0.45	1.00	0.12	0.94
11	I use SNS longer than I intend to.	3.81	0.40	1.00	0.10	1.00
12	I tell myself to stop using SNS, but I keep doing it.	3.88	0.34	1.00	0.09	1.00
13	Once I start using SNS, I can’t stop.	3.88	0.34	1.00	0.09	1.00
	Withdrawal sub-domain					
14	I feel anxious and restless when I reduce or stop using SNS.	3.81	0.40	1.00	0.10	1.00
15	I feel irritated when I reduce or stop using SNS.	3.81	0.40	1.00	0.10	1.00
16	I get headaches when I reduce or stop using SNS.	3.69	0.60	0.88	0.16	0.94
17	My appetite increases or decreases when I reduce or stop using SNS.	3.63	0.62	0.88	0.17	0.75
	Relapse sub-domain					
18	I have tried to quit using SNS but find it difficult and end up using it again.	3.81	0.40	1.00	0.10	1.00
19	When I quit SNS, I cannot resist the urge to use it again.	3.75	0.45	1.00	0.12	0.94
20	After quitting SNS, I find it hard to suppress the desire to use it when I see others using it.	3.69	0.48	1.00	0.13	0.75
	Education/work problems sub-domain					
21	I sometimes miss important academic or work-related tasks because of using SNS.	3.94	0.25	1.00	0.06	1.00
22	My academic performance or work productivity declines due to SNS usage.	3.88	0.34	1.00	0.09	1.00
23	Frequent thoughts about SNS disrupt my academic or work activities.	3.94	0.25	1.00	0.06	1.00
24	I don’t have time for my studies or work because I spend too much time on SNS.	3.88	0.34	1.00	0.09	1.00
	Health management issues sub-domain					
25	My excessive SNS use hinders the formation of healthy habits.	3.81	0.40	1.00	0.10	1.00
26	I have missed exercises because of using SNS.	3.88	0.34	1.00	0.09	1.00
27	I have hastily eaten meals due to using SNS.	3.81	0.40	1.00	0.10	1.00
28	I feel inferior when I see others’ posts on SNS.	3.94	0.25	1.00	0.06	1.00
29	If my posts get few reactions, I wonder if what I posted wasn’t good.	3.88	0.34	1.00	0.09	1.00
	Rest and sleep problems sub-domain					
30	I lack sleep because I spend too much time on SNS.	3.88	0.34	1.00	0.09	1.00
31	I go to bed later because of using SNS.	3.81	0.40	1.00	0.10	1.00
32	My sleep patterns become irregular because of SNS.	3.88	0.34	1.00	0.09	1.00
33	I lack relaxation time due to using SNS.	3.94	0.25	1.00	0.06	1.00
34	I cannot rest my body and mind properly because of SNS.	3.81	0.40	1.00	0.10	1.00
	Social relationship issues sub-domain					
35	I have ignored what others were saying because I was using SNS.	3.88	0.34	1.00	0.09	1.00
36	I have lost important relationships due to being overly absorbed in SNS.	3.94	0.25	1.00	0.06	1.00
37	My use of SNS causes conflicts with my friends.	3.88	0.34	1.00	0.09	1.00
38	I feel closer to my SNS friends than to my family or friends.	3.88	0.34	1.00	0.09	1.00
39	I feel that my SNS friends understand me better than my family or friends do.	3.94	0.25	1.00	0.06	1.00
	Social participation issues sub-domain					
40	I meet people more through SNS than in real life.	3.88	0.34	1.00	0.09	1.00
41	I spend less time with friends because of SNS usage.	3.94	0.25	1.00	0.06	1.00
42	I spend less time and participate less frequently in various events, activities, and gatherings because of using SNS.	3.88	0.34	1.00	0.09	1.00

**Table 7:** CVR, Stability, and Consensus Results for All Delphi Rounds.

	M*	SD**	CVR***	Stability	Consensus
**Domain**					
Round 1	3.72	0.49	0.91	0.14	0.90
Round 2	3.71	0.46	0.99	0.12	0.88
Round 3	3.81	0.40	1.00	0.10	0.98
**Item**					
Round 1	3.57	0.60	0.83	0.18	0.78
Round 2	3.69	0.53	0.94	0.19	0.88
Round 3	3.83	0.38	0.99	0.10	0.97

* M = mean;

** SD = standard deviation;

*** CVR = Content Validity Ratio.

## Discussion

This study developed a questionnaire for the PSNSU targeting adolescents and young adults and verified its content validity using the Delphi survey methodology. In the final round of the Delphi survey, the PSNSU questionnaire demonstrated high content validity, with an average CVR of 1.00, stability of 0.10, consensus of 0.98, CVR of 0.99, stability of 0.10, and consensus of 0.97, indicating strong expert agreement on the relevance of the questionnaire content.

The multidomain structure of the PSNSU questionnaire validated in this study allows for a more holistic understanding of PSNSU. Previous research has primarily concentrated on addictive aspects as a single domain [16,17], neglecting the broader effects on everyday life. Addictive elements, such as mood modification, salience, tolerance, withdrawal, and relapse, which were not deleted by Delphi, have been extensively discussed in the context of substance addiction [34]. However, assessing digital addiction in modern society using only these addictive elements is limited [35,36] because it overlooks the multifaceted nature of PSNSU, which extends beyond addiction and impacts daily functioning and social interactions. Previous studies [19,37] reported that it is more appropriate to approach this issue from the perspective of problematic use rather than within an addiction framework to avoid misdiagnosing problematic use as addiction. This study fills this gap by developing a PSNSU assessment tool that not only evaluates addictive behaviors but also addresses associated everyday problems such as occupational difficulties and social issues, thereby providing a comprehensive measure of the impact of PSNSU on an individual’s life.

Through the Delphi process, the key domains were defined as addictive and occupational problems. Initially, the questionnaire included a social problem domain; however, it was consolidated into an occupational problem domain after obtaining expert consensus. Specifically, the SNS-centered social network, peer phubbing subdomains, and items from the social problem domain were moved to the social relationships and social participation problem subdomains within the occupational problem domain, providing a more cohesive structure. This adjustment demonstrated a high level of expert consensus and was aligned with the existing framework, which broadly describes occupational issues as those related to daily living activities [20].

Among the two domains, the occupational problem domain is particularly noteworthy, as it has been largely overlooked in the existing PSNSU scales and assessments. By including the occupational problem domain, SNS should be regarded as part of the daily lifestyle [11], as the tools can better capture the influences that are deeply related to the user’s everyday life. Subdomains such as education/work problems have been included in previous assessment tools [17,38], but they are often measured with a single item, limiting the ability to form a comprehensive factor. In this study, each subdomain is composed of at least three items, which is the recommended number of items per domain in a questionnaire [39,40]. This allowed for the evaluation of the comprehensive aspects of the PSNSU and facilitated the application of more systematic evaluations related to the user’s everyday life impact.

This study has several advantages. First, we developed a multidimensional tool for assessing PSNSU, providing a comprehensive understanding beyond the single-dimensional approach. This tool is significant because it evaluates not only the addictive aspects of PSNSU but also its impact on occupational problems in everyday life, which were previously unassessed by other tools. Second, the questionnaire targets both adolescents and adults, making it useful for measuring, tracking, and analyzing developing adolescents and adults using a single tool. This allows for age-specific assessments and provides data for tailoring intervention strategies. Third, the content validity of the questionnaire has been proven by a wide range of experts, making it applicable for systematically evaluating and managing PSNSU in various settings such as schools, workplaces, and communities, in academic research and practical environments.

This study has several limitations. First, it did not include a pilot testing phase with actual users, which was crucial for validating the practical applicability and reliability of the questionnaire. Previous studies [41,42] indicated that online connections can be healthier and more positive for users whose sexual preferences or gender identity is not accepted by their community. Therefore, future studies should ensure the validity of the PSNSU by clarifying the target study group and collecting data from a representative user population for comprehensive analysis. Second, the questionnaire developed in this study relies mainly on self-reporting, which may lead to a response bias based on the respondents’ subjective perceptions. To complement this, future research should include objective data (e.g., SNS usage time and usage patterns). Additionally, because of the nature of SNS, problematic behaviors may vary by platform [43,44]. However, this scale evaluates only broad SNS use, which may be insufficient for obtaining information about specific platforms or behaviors. Future research should identify response differences in the PSNSU questionnaire based on the SNS platforms used by the respondents. Third, although the content validity of this evaluation tool was verified through a Delphi study, the item structure of the PSNSU evaluation tool was not statistically confirmed. Therefore, confirmatory factor analysis and Receiver Operating Characteristic analysis are needed using data from a representative sample to verify whether the PSNSU questionnaire structures two domains—–10 subdomains and 42 items—–and has an appropriate response scale. In addition, the reliability and validity of the evaluation tool must be verified.

## Conclusion

The final PSNSU questionnaire for adolescents and adults includes two domains and ten subdomains, encompassing a wide range of issues related to addictive behaviors and occupational challenges. The development and analysis of the PSNSU questionnaire using the Delphi survey methodology validated the content relevance of the two aspects of the PSNSU. By addressing the various dimensions of problematic SNS use and incorporating expert feedback, this questionnaire provides a valuable resource for both research and clinical practice, aiding in the identification and management of PSNSU across different life stages.

## Supporting information

S1 File Raw Data Collected in All Delphi Rounds. (XLSX)

S1 Table Problematic Social network Sites Usage Questionnaire with Confirmed Content Validity. (DOCX)

## References

[1] Laghari AA, He H, Shafiq M, Khan A. Application of quality of experience in networked services: review, trend & perspectives. Syst Pract Action Res. 2018;32(5):501–19. doi: 10.1007/s11213-018-9471-x

[2] Tecson-Turano C. Social media as a communication tool to support university student - services: affordances, limitations and opportunities for innovations. IJICS. 2017;2(5):75. doi: 10.11648/j.ijics.20170205.14

[3] Statista.com. Number of social media users worldwide from 2010 to 2021 (in billions). הארץ. 2022;2021:2003–5.

[4] Whiting A, Williams D. Why people use social media: a uses and gratifications approach. Qual Market Res: An Int J. 2013;16(4):362–9. doi: 10.1108/qmr-06-2013-0041

[5] Hussain Z, Griffiths MD. Problematic social networking site use and comorbid psychiatric disorders: a systematic review of recent large-scale studies. Front Psychiatry. 2018;9:686. doi: 10.3389/fpsyt.2018.00686 30618866 PMC6302102

[6] Andreassen CS, Torsheim T, Brunborg GS, Pallesen S. Development of a facebook addiction scale. Psychol Rep. 2012;110(2):501–17. doi: 10.2466/02.09.18.PR0.110.2.501-517 22662404

[7] Kuss DJ, Griffiths MD. Online social networking and addiction--a review of the psychological literature. Int J Environ Res Public Health. 2011;8(9):3528–52. doi: 10.3390/ijerph8093528 22016701 PMC3194102

[8] Steinberger P, Kim H. Social comparison of ability and fear of missing out mediate the relationship between subjective well-being and social network site addiction. Front Psychol. 2023;14:1157489. doi: 10.3389/fpsyg.2023.1157489 37434890 PMC10332318

[9] Chu X, Ji S, Wang X, Yu J, Chen Y, Lei L. Peer phubbing and social networking site addiction: the mediating role of social anxiety and the moderating role of family financial difficulty. Front Psychol. 2021;12:670065. doi: 10.3389/fpsyg.2021.670065 34421727 PMC8374054

[10] Ciudad-Fernández V, Zarco-Alpuente A, Escrivá-Martínez T, Herrero R, Baños R. How adolescents lose control over social networks: a process-based approach to problematic social network use. Addict Behav. 2024;154:108003. doi: 10.1016/j.addbeh.2024.108003 38461744

[11] Hylkilä K, Männikkö N, Peltonen A, Castrén S, Mustonen T, Konttila J, et al. Association between problematic social networking site use and social well-being among young adults: a systematic review. J Affect Disord Reps. 2024;16:100775. doi: 10.1016/j.jadr.2024.100775

[12] Liu L, Yao Y-W, Li C-SR, Zhang J-T, Xia C-C, Lan J, et al. The comorbidity between internet gaming disorder and depression: interrelationship and neural mechanisms. Front Psychiatry. 2018;9:154. doi: 10.3389/fpsyt.2018.00154 29740358 PMC5924965

[13] Jeong H, Yim HW, Lee S-Y, Lee HK, Potenza MN, Jo S-J, et al. Reciprocal relationship between depression and Internet gaming disorder in children: A 12-month follow-up of the iCURE study using cross-lagged path analysis. J Behav Addict. 2019;8(4):725–32. doi: 10.1556/2006.8.2019.74 32359239 PMC7044588

[14] Pang H, Ji M, Hu X. How differential dimensions of social media overload influences young people’s fatigue and negative coping during prolonged COVID-19 pandemic? Insights from a technostress perspective. Healthcare (Basel). 2022;11(1):6. doi: 10.3390/healthcare11010006 36611466 PMC9818937

[15] Seabrook EM, Kern ML, Rickard NS. Social networking sites, depression, and anxiety: a systematic review. JMIR Ment Health. 2016;3(4):e50. doi: 10.2196/mental.5842 27881357 PMC5143470

[16] Schou Andreassen C, Billieux J, Griffiths MD, Kuss DJ, Demetrovics Z, Mazzoni E, et al. The relationship between addictive use of social media and video games and symptoms of psychiatric disorders: a large-scale cross-sectional study. Psychol Addict Behav. 2016;30(2):252–62. doi: 10.1037/adb0000160 26999354

[17] van den Eijnden RJJM, Lemmens JS, Valkenburg PM. The social media disorder scale. Comput Human Behav. 2016;61 478–87. doi: 10.1016/j.chb.2016.03.038

[18] Wadsley M. A multi-modal investigation of reward-based mechanisms underlying excessive and problematic social networking site use. 2023. 10.1177/00332941211025271 PMC9483697 34162237

[19] Panova T, Carbonell X. Is smartphone addiction really an addiction? J Behav Addict. 2018;7(2):252–9. doi: 10.1556/2006.7.2018.49 29895183 PMC6174603

[20] Boop C, Cahill SM, Davis C. Occupational therapy practice framework: domain and process-fourth edition. Am J Occup Ther. 2020;74(Supplement_2):7412410010p1–87. doi: 10.5014/ajot.2020.74S2001 34780625

[21] González-Nuevo C, Cuesta M, Postigo Á, Menéndez-Aller Á, Muñiz J. Problematic social network use: structure and assessment. Int J Ment Health Addict. 2021;1–16. doi: 10.1007/s11469-021-00711-y 34876890 PMC8638649

[22] Obeid S, González-Nuevo C, Postigo Á, El Dine AS, Azzi V, Malaeb D, et al. Psychometric properties of the Problematic Use of Social Networks (PUS) scale in Arabic among adolescents. PLoS One. 2023;18(9):e0291616. doi: 10.1371/journal.pone.0291616 37708202 PMC10501610

[23] Sangchooli A, Hamzehzadeh M, Rafiemanesh H, Ghani K, Farnam R, Rahimi-Movaghar A. Development and psychometric evaluation of a DSM-V-based social networking site use disorder test: the SNS-DT-10. Psychol Rep. 2023;126(3):1551–62. doi: 10.1177/00332941211065950 35067129

[24] Li Q-Q, Lee J-H, Kim E-M, Bak B-G. Development and validation of smartphone addiction scale for college students. KJCE. 2021;30(1):157–84. doi: 10.17643/kjce.2021.30.1.08

[25] Kim J-N. Development and validation of SNS addiction proneness scale for college students. Korean J Health Psychol. 2014;19(1):147–66. doi: 10.17315/KJHP.2014.19.1.008

[26] Lynn MR. Determination and quantification of content validity. Nurs Res. 1986;35(6):382–5. doi: 10.1097/00006199-198611000-00017 3640358

[27] Dalkey N, Helmer O. An experimental application of the DELPHI method to the use of experts. Manag Sci. 1963;9(3):458–67. doi: 10.1287/mnsc.9.3.458

[28] Linstone HA, Turoff M. The Delphi method: Techniques and applications. Delphi Method Book; 2002.

[29] Rowe G, Wright G. The Delphi technique as a forecasting tool. Int J Forecast. 1999;15:353–75.

[30] Lawshe C. A quantitative approach to content validity. Pers Psychol. 1975;28:563–75.

[31] Dajani JS, Sincoff MZ, Talley WK. Stability and agreement criteria for the termination of Delphi studies. Technol Forecast Soc Change. 1979;13(1):83–90. doi: 10.1016/0040-1625(79)90007-6

[32] Hsu C, Sandford B. The Delphi technique: making sense of consensus. Pract Assessment, Res Eval. n.d.;12(1):1–8.

[33] Diamond IR, Grant RC, Feldman BM, Pencharz PB, Ling SC, Moore AM, et al. Defining consensus: a systematic review recommends methodologic criteria for reporting of Delphi studies. J Clin Epidemiol. 2014;67(4):401–9. doi: 10.1016/j.jclinepi.2013.12.002 24581294

[34] Griffiths M. A ‘components’ model of addiction within a biopsychosocial framework. J Substance Use. 2005;10(4):191–7. doi: 10.1080/14659890500114359

[35] Igbashangev P, Abdullahi S, Terungwa BA, et al. Social media as a tool for the advancement of democracy and good governance in Nigeria. Soc Sci Res Netw. 2023.

[36] Collings B. Selfie to healthy: designing behaviour change programs for young women to healthily engage with social media selfie filters. 2023.

[37] Tobin SJ, Graham S. Feedback sensitivity as a mediator of the relationship between attachment anxiety and problematic facebook use. Cyberpsychol Behav Soc Netw. 2020;23(8):562–6. doi: 10.1089/cyber.2019.0560 32478571

[38] Kang H, Park C. Development and validation of the Smartphone Addiction Inventory. Korean J Psychol. 2012;31(1):2012.

[39] Marsh HW, Hau KT, Balla JR, Grayson D. Is more ever too much? The Number of indicators per factor in confirmatory factor analysis. Multivariate Behav Res. 1998;33(2):181–220. doi: 10.1207/s15327906mbr3302_1 26771883

[40] DeVellis RF, Thorpe CT. Scale development: Theory and applications. 2021.

[41] Craig SL, Eaton AD, McInroy LB, Leung VWY, Krishnan S. Can social media participation enhance LGBTQ+ youth well-being? Development of the social media benefits scale. Social Media + Society. 2021;7(1). doi: 10.1177/2056305121988931

[42] Pellicane MJ, Cooks JA, Ciesla JA. Longitudinal effects of social media experiences on depression and anxiety in LGB+ and heterosexual young adults. J Gay Lesbian Mental Health. 2020;25(1):68–93. doi: 10.1080/19359705.2020.1776805

[43] Bayer JB, Anderson IA, Tokunaga RS. Building and breaking social media habits. Curr Opin Psychol. 2022;45:101303. doi: 10.1016/j.copsyc.2022.101303 35255413

[44] Vaghefi I, Negoita B, Lapointe L. The path to hedonic information system use addiction: a process model in the context of social networking sites. Inform Syst Res. 2023;34(1):85–110. doi: 10.1287/isre.2022.1109

