# Supplementary material for: Development of a questionnaire for problematic social networking sites use: Ensuring content validity through Delphi methodology
Source: PLoS One. 2025 Mar 10;20(3):e0315442. doi: 10.1371/journal.pone.0315442 (PMC11892839; doi:10.1371/journal.pone.0315442)
Supplement: S1 Table — (DOCX) [file pone.0315442.s002.docx]

**S1 Table.** **Problematic Social Network Sites (SNS) Usage questionnaire**

| Items | | Answer | | | | |  |
| --- | --- | --- | --- | --- | --- | --- | --- |
|  |  | [1] Very Disagree ~ [5] Very Agree | | | | |  |
| 1 | I use SNS for a change of mood. | 1 | 2 | 3 | 4 | 5 | |
| 2 | I use SNS to forget about troublesome matters. | 1 | 2 | 3 | 4 | 5 | |
| 3 | I use SNS to reduce depression and anxiety. | 1 | 2 | 3 | 4 | 5 | |
| 4 | I use SNS to feel calm and stable. | 1 | 2 | 3 | 4 | 5 | |
| 5 | I think about SNS even when I am not using it. | 1 | 2 | 3 | 4 | 5 | |
| 6 | I repeatedly think about things I saw on SNS. | 1 | 2 | 3 | 4 | 5 | |
| 7 | I find it difficult to resist the urge to use SNS. | 1 | 2 | 3 | 4 | 5 | |
| 8 | I frequently want to check SNS notifications while doing other tasks. | 1 | 2 | 3 | 4 | 5 | |
| 9 | I spend more and more time on SNS. | 1 | 2 | 3 | 4 | 5 | |
| 10 | No matter how much I use SNS, I feel it's not enough. | 1 | 2 | 3 | 4 | 5 | |
| 11 | I use SNS longer than I intend to. | 1 | 2 | 3 | 4 | 5 | |
| 12 | I tell myself to stop using SNS, but I keep doing it. | 1 | 2 | 3 | 4 | 5 | |
| 13 | Once I start using SNS, I can't stop. | 1 | 2 | 3 | 4 | 5 | |
| 14 | I feel anxious and restless when I reduce or stop using SNS. | 1 | 2 | 3 | 4 | 5 | |
| 15 | I feel irritated when I reduce or stop using SNS. | 1 | 2 | 3 | 4 | 5 | |
| 16 | I get headaches when I reduce or stop using SNS. | 1 | 2 | 3 | 4 | 5 | |
| 17 | My appetite increases or decreases when I reduce or stop using SNS. | 1 | 2 | 3 | 4 | 5 | |
| 18 | I have tried to quit using SNS but find it difficult and end up using it again. | 1 | 2 | 3 | 4 | 5 | |
| 19 | When I quit SNS, I cannot resist the urge to use it again. | 1 | 2 | 3 | 4 | 5 | |
| 20 | After quitting SNS, I find it hard to suppress the desire to use it when I see others using it. | 1 | 2 | 3 | 4 | 5 | |
| 21 | I sometimes miss important academic or work-related tasks because of using SNS. | 1 | 2 | 3 | 4 | 5 | |
| 22 | My academic performance or work productivity declines due to SNS usage. | 1 | 2 | 3 | 4 | 5 | |
| 23 | Frequent thoughts about SNS disrupt my academic or work activities. | 1 | 2 | 3 | 4 | 5 | |
| 24 | I don't have time for my studies or work because I spend too much time on SNS. | 1 | 2 | 3 | 4 | 5 | |
| 25 | My excessive SNS use hinders the formation of healthy habits. | 1 | 2 | 3 | 4 | 5 | |
| 26 | I have missed exercises because of using SNS. | 1 | 2 | 3 | 4 | 5 | |
| 27 | I have hastily eaten meals due to using SNS. | 1 | 2 | 3 | 4 | 5 | |
| 28 | I feel inferior when I see others' posts on SNS. | 1 | 2 | 3 | 4 | 5 | |
| 29 | If my posts get few reactions, I wonder if what I posted wasn't good. | 1 | 2 | 3 | 4 | 5 | |
| 30 | I lack sleep because I spend too much time on SNS. | 1 | 2 | 3 | 4 | 5 | |
| 31 | I go to bed later because of using SNS. | 1 | 2 | 3 | 4 | 5 | |
| 32 | My sleep patterns become irregular because of SNS. | 1 | 2 | 3 | 4 | 5 | |
| 33 | I lack relaxation time due to using SNS. | 1 | 2 | 3 | 4 | 5 | |
| 34 | I cannot rest my body and mind properly because of SNS. | 1 | 2 | 3 | 4 | 5 | |
| 35 | I have ignored what others were saying because I was using SNS. | 1 | 2 | 3 | 4 | 5 | |
| 36 | I have lost important relationships due to being overly absorbed in SNS. | 1 | 2 | 3 | 4 | 5 | |
| 37 | My use of SNS causes conflicts with my friends. | 1 | 2 | 3 | 4 | 5 | |
| 38 | I feel closer to my SNS friends than to my family or friends. | 1 | 2 | 3 | 4 | 5 | |
| 39 | I feel that my SNS friends understand me better than my family or friends do. | 1 | 2 | 3 | 4 | 5 | |
| 40 | I meet people more through SNS than in real life. | 1 | 2 | 3 | 4 | 5 | |
| 41 | I spend less time with friends because of SNS usage. | 1 | 2 | 3 | 4 | 5 | |
| 42 | I spend less time and participate less frequently in various events, activities, and gatherings because of using SNS. | 1 | 2 | 3 | 4 | 5 | |
